# Thalamic pathology and memory loss in early Alzheimer’s disease: moving the focus from the medial temporal lobe to Papez circuit

**DOI:** 10.1093/brain/aww083

**Published:** 2016-04-28

**Authors:** John P. Aggleton, Agathe Pralus, Andrew J. D. Nelson, Michael Hornberger

**Affiliations:** ^1^ School of Psychology, Cardiff University, Park Place, Cardiff, CF10 3AT, UK; ^2^ Master of Biosciences, ENS de Lyon, 46 allée d'Italie, 69007 Lyon, France; ^3^ Norwich Medical School, University of East Anglia, Norwich, NR4 7TJ, UK

**Keywords:** anterior thalamic nuclei, dementia, limbic thalamus, memory, retrosplenial cortex

## Abstract

It is widely assumed that incipient protein pathology in the medial temporal lobe instigates the loss of episodic memory in Alzheimer’s disease, one of the earliest cognitive deficits in this type of dementia. Within this region, the hippocampus is seen as the most vital for episodic memory. Consequently, research into the causes of memory loss in Alzheimer’s disease continues to centre on hippocampal dysfunction and how disease-modifying therapies in this region can potentially alleviate memory symptomology. The present review questions this entrenched notion by bringing together findings from post-mortem studies, non-invasive imaging (including studies of presymptomatic, at-risk cases) and genetically modified animal models. The combined evidence indicates that the loss of episodic memory in early Alzheimer’s disease reflects much wider neurodegeneration in an extended mnemonic system (Papez circuit), which critically involves the limbic thalamus. Within this system, the anterior thalamic nuclei are prominent, both for their vital contributions to episodic memory and for how these same nuclei appear vulnerable in prodromal Alzheimer’s disease. As thalamic abnormalities occur in some of the earliest stages of the disease, the idea that such changes are merely secondary to medial temporal lobe dysfunctions is challenged. This alternate view is further strengthened by the interdependent relationship between the anterior thalamic nuclei and retrosplenial cortex, given how dysfunctions in the latter cortical area provide some of the earliest *in vivo* imaging evidence of prodromal Alzheimer’s disease. Appreciating the importance of the anterior thalamic nuclei for memory and attention provides a more balanced understanding of Alzheimer’s disease. Furthermore, this refocus on the limbic thalamus, as well as the rest of Papez circuit, would have significant implications for the diagnostics, modelling, and experimental treatment of cognitive symptoms in Alzheimer’s disease.

## Teaser

Research into episodic memory loss in Alzheimer’s disease has repeatedly focused on the hippocampus. Aggleton et al. argue that this approach is too narrow, and ignores the early involvement of other brain sites, most notably the anterior thalamic nuclei, which are also vital for episodic memory.

## Graphical abstract

**Figure:**
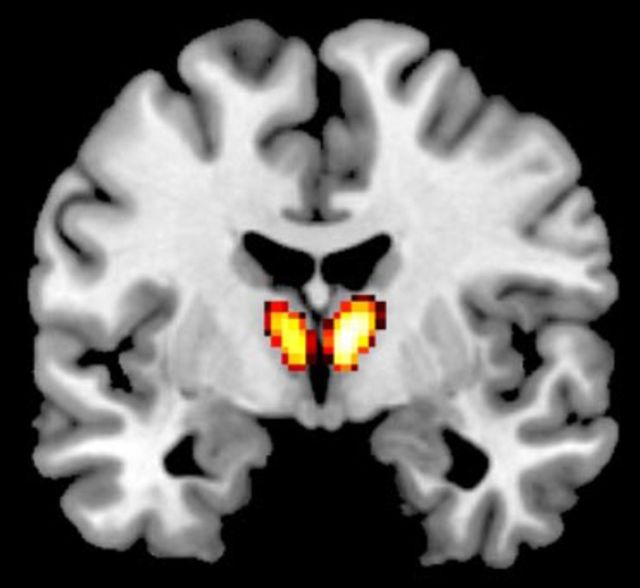
Research into episodic memory loss in Alzheimer’s disease has repeatedly focused on the hippocampus. Aggleton et al. argue that this approach is too narrow, and ignores the early involvement of other brain sites, most notably the anterior thalamic nuclei, which are also vital for episodic memory.

## Introduction

Alzheimer’s disease is the most prevalent form of dementia, currently affecting 47 million people worldwide, with the number of patients with Alzheimer’s disease projected to triple over the next 30 years ( WHO, 2012 ). There is an urgent need for disease-modifying therapies to alleviate or, ideally, halt Alzheimer’s disease symptoms. While therapies are currently in development, the effectiveness of this endeavour is critically dependent on sensitive outcome measures that target regional specific dysfunctions in Alzheimer’s disease. Most outcome measures concern the ability to remediate the well-established pathological changes seen in the medial temporal lobe in Alzheimer’s disease and its prodromal forms ( Hyman *et al.* , 1990 ; Braak *et al.* , 1997 ; Craig *et al.* , 2011 ). This attention on the medial temporal lobe is reinforced by the long-standing assumption that the hippocampus, in concert with its parahippocampal connections, is the pre-eminent brain region supporting episodic memory ( Spiers *et al.* , 2001 ; Squire *et al.* , 2004 ; Diana *et al.* , 2007 ), the category of memory that concerns the day-to-day events that make up our lives. In this way, neuropathological findings from patients with Alzheimer’s disease have been integrated with its leading cognitive failure (episodic memory loss), further adding to the focus on the medial temporal lobe. Consequently, it has been argued that an amnesic syndrome of the hippocampal type is an essential core feature for the diagnosis of typical Alzheimer’s disease ( Sarazin *et al.* , 2007 ). Indeed, Alzheimer’s disease has been seen by some as fundamentally a hippocampal dementia ( Craig *et al.* , 2011 ). In practice, Alzheimer’s disease can also present with prominent language (e.g. logopenic aphasia), visual (e.g. posterior cortical atrophy) or motor (e.g. corticobasal degeneration) symptoms, reflecting different combinations of pathologies that extend beyond the hippocampus ( Hof *et al.* , 1997 ; Caine and Hodges, 2001 ; Lambon Ralph *et al.* , 2003 ; Dubois *et al.* , 2010 ).

**Figure 1:**
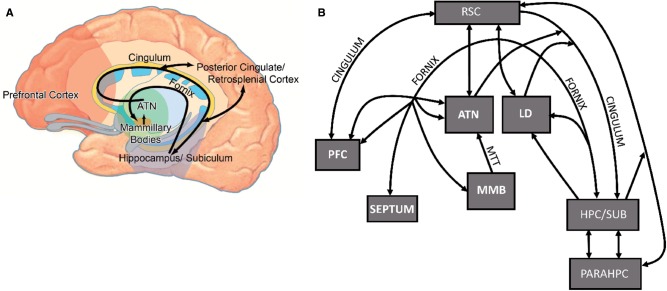
**Illustrations showing the location of the anterior thalamic nuclei within Papez circuit and the limbic system.** ( **A** ) Schematic drawing of Papez circuit (in black). ( **B** ) Schematic diagram of the key connections between the anterior thalamic nuclei and laterodorsal thalamic nucleus with sites implicated in Alzheimer’s disease. ATN = anterior thalamic nuclei; HPC/SUB = hippocampal formation, including subiculum; LD = laterodorsal thalamic nucleus; MMB = mammillary bodies; MTT = mammillothalamic tract; PARAHPC = parahippocampal region; PFC = prefrontal cortex; RSC = retrosplenial cortex.

While the continuing focus on the medial temporal lobe for memory loss is understandable, it comes at a potential price. There remains the possibility that pathologies in other areas play a key role in disrupting memory, starting from the earliest stages of the disease. Two candidate areas are: (i) the posterior cingulate region, which includes the retrosplenial cortex (see ‘Anterior thalamic–retrosplenial cortex interactions’ section); and (ii) the limbic thalamus. The principal nuclei of the limbic thalamus consist of the anterior thalamic nuclei, the lateral dorsal nucleus, and the medial dorsal nucleus ( Taber *et al.* , 2004 ). Both the posterior cingulate region and the anterior thalamic nuclei are key components of Papez circuit ( Fig. 1 ), so that both have dense, direct hippocampal connections, which principally involve the subiculum and presubiculum ( Aggleton *et al.* , 1986 ; Vogt *et al.* , 1987 ; Kobayashi and Amaral, 2003 , 2007 ; Aggleton, 2012 ).

There has been a steady growth of interest in the potential importance of the posterior cingulate region during the initial development of Alzheimer’s disease (see below). As noted, its apparent importance could reflect the many direct, reciprocal connections between the posterior cingulate cortices and the hippocampal formation ( Vogt *et al.* , 1987 ; Kobayashi and Amaral, 2003 , 2007 ; Aggleton *et al.* , 2014 ). Alternatively, it could reflect how the posterior cingulate region is part of Papez circuit ( Fig. 1 ), where it forms a node between the anterior thalamic nuclei and the hippocampal formation ( Vann *et al.* , 2009 ). To distinguish these accounts it is necessary to examine the status of specific thalamic nuclei during the development of Alzheimer’s disease. If it appears that anterior thalamic dysfunction does not contribute to the early stages of Alzheimer’s disease, then it can be assumed that it is the direct hippocampal–posterior cingulate connections that suffer the key, initial dysfunctions. If, however, anterior thalamic dysfunctions are present from the earliest stages of the disorder, this calls for a systems analysis that involves the wider (Papez) network. Unfortunately, the thalamus has received relatively little attention with respect to Alzheimer’s disease.

Among the various groups of thalamic nuclei, the limbic thalamus stands out by virtue of its importance for cognition ( Vogt and Gabriel, 1993 ; Aggleton and Brown, 1999 ). Within this group, the anterior thalamic nuclei appear to show the clearest structural changes with normal ageing ( Fama and Sullivan, 2015 ). In addition, anterior thalamic dysfunctions are a core, contributing feature of diencephalic amnesia, which is characterized by a loss of episodic memory ( Aggleton and Brown, 1999 ; Harding *et al.* , 2000 ; Van der Werf *et al.* , 2003 ; Carlesimo *et al.* , 2011 ). Consequently, abnormalities in these nuclei have the potential to cause profound changes in cognition. The anterior thalamic nuclei also stand out as, along with the laterodorsal thalamic nucleus, they have dense, reciprocal connections with both the hippocampal formation and retrosplenial cortex ( Amaral and Cowan, 1980 ; Aggleton *et al.* , 1986 ; Vogt *et al.* , 1987 ; Xiao and Barbas, 2002 *a* , *b* ). Furthermore, animal studies show how both anterior thalamic–hippocampal and anterior thalamic–retrosplenial interconnections support aspects of episodic memory ( Sutherland and Hoesing, 1993 ; Parker and Gaffan, 1997 ; Warburton *et al.* , 2001 ; Henry *et al.* , 2004 ).

Taken together, clinical and behavioural findings indicate that interactions along Papez circuit ( Fig. 1 A), which involve the hippocampus, fornix, mammillary bodies, anterior thalamic nuclei, and posterior cingulate region, are critical for episodic memory ( Delay and Brion, 1969 ; Aggleton and Brown, 1999 ; Tsivilis *et al.* , 2008 ; Carlesimo *et al.* , 2011 ). Consequently, this limbic network could be of especial relevance when considering the diagnosis and potential treatment of Alzheimer’s disease.

The present review examines how thalamic changes might contribute to Alzheimer’s disease symptomology. The least contentious proposal is that medial temporal lobe neuropathologies instigate dysfunctions across an interconnected network of medial temporal, thalamic, and parietal sites, thereby providing a more complete explanation of the ways in which Alzheimer’s disease and its prodromal stages affect memory. A more radical model is that thalamic neuropathologies occur at the same time, or even before, those in the medial temporal lobe. Consequently, thalamic dysfunctions may contribute or even be responsible for some of the earliest cognitive symptoms of mild cognitive impairment (MCI) and Alzheimer’s disease. In comparing these scenarios we will consider whether medial temporal lobe pathology is sufficient to explain memory loss in Alzheimer’s disease or whether thalamic pathology is a necessary feature.

Reflecting these various goals, the review first considers post-mortem evidence, as this provides the necessary anatomical resolution to distinguish individual thalamic nuclei. Next, non-invasive imaging findings from Alzheimer’s disease and its prodromal forms are described, including findings from presymptomatic, at-risk patients. Complementary evidence from animal models of dementia is then described, which offers a way to isolate early disease processes. Finally, evidence is provided from experimental interventions that point to the importance of anterior thalamic actions upon the medial temporal lobe, including the hippocampus.

## Thalamic neuropathology in Alzheimer’s disease

In their landmark paper, Braak and Braak (1991 *a* ) described the chronological staging of neuropathological changes in Alzheimer’s disease. Their focus was on extracellular amyloid (e.g. plaques) and intraneuronal neurofibrillary changes (e.g. tau tangles and neuropil threads). While amyloid deposition was variable, the pattern of neurofibrillary changes provided a more consistent sequence, beginning in the entorhinal region (Stages I–II). Although Braak and Braak (1991 *a* ) emphasized the involvement of the hippocampus (Stages III–IV) they examined many other areas. Of these other areas, the anterodorsal thalamic nucleus stood out by having marked neurofibrillary changes at the same time as the hippocampus (Stages III–IV), i.e. prior to many other sites ( Braak and Braak, 1991 *a* ).

**Figure 2:**
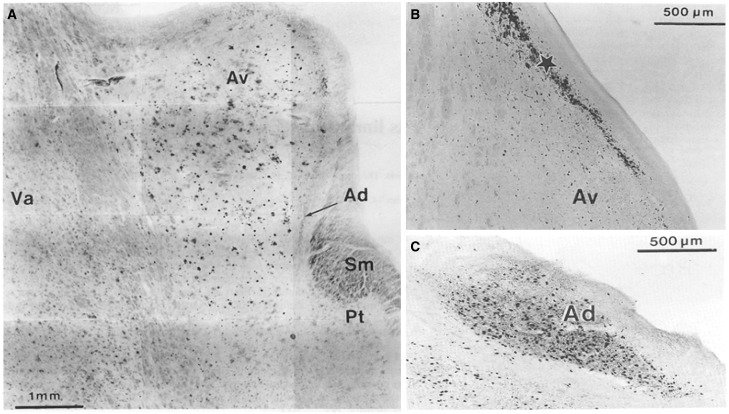
**Post-mortem pathology in the anterior thalamus in patients with Alzheimer’s disease.** The coronal images are taken with permission from Braak and Braak (1991 *b* ). ( **A** ) Conspicuous patches of amyloid deposition in the anteroventral thalamic nucleus. ( **B** ) Presence of neurofibrillary changes adjacent to the anteroventral thalamic nucleus. ( **C** ) Dense deposition of neurofibrillary tangles in the anterodorsal thalamic nucleus. Av = anteroventral thalamic nucleus; Ad = anterodorsal thalamic nucleus; Pt = parataenial nucleus; SM = stria medullaris; Va = ventral anterior nucleus.

A second report just considered the thalamus in post-mortem Alzheimer’s disease brains ( Braak and Braak, 1991 *b* ). While patches of amyloid were present across numerous thalamic nuclei, neurofibrillary changes were far more restricted ( Fig. 2 ). Many nuclei appeared largely unaffected or showed only mild neurofibrillary changes, even in cases of severe Alzheimer’s disease ( Braak and Braak, 1991 *b* ). Nevertheless, some thalamic nuclei (anterodorsal, anteroventral, laterodorsal, central medial, parataenial, and reticular nuclei) displayed conspicuous neurofibrillary deposits ( Fig. 2 ). These nuclei contrasted with the adjacent medial dorsal nucleus, which appeared unaffected in their study. As noted above, the anterodorsal nucleus was most affected, being ‘infested’ with neurofibrillary tangles ( Braak and Braak, 1991 *b* ; see also Xuereb *et al.* , 1991 ). Dense neurofibrillary changes were also found between the anteroventral nucleus and the ependymal lining of the third ventricle. The patterns of neuropathology in the laterodorsal nucleus closely mirrored those in the anteroventral nucleus. By the later stages (V and VI), neurofibrillary changes were also prominent in nucleus reuniens and the reticular thalamic nucleus, but these nuclei lacked amyloid patches ( Braak and Braak, 1991 *b* ). This distribution of tau pathology in the later stages of Alzheimer’s disease, i.e. focused in the anterodorsal, laterodorsal, and paraventricular nuclei of the limbic thalamus, has since been confirmed ( Rüb *et al.* , 2015 ).

This pattern of thalamic neurofibrillary change is striking as it preferentially maps onto those nuclei with reciprocal hippocampal connections ( Fig. 1 B). Hippocampal inputs predominantly reach the thalamus via the fornix ( Aggleton, 2012 ), a tract that atrophies in Alzheimer’s disease and its prodromal stages ( Hooper and Vogel, 1976 ; Metzler-Baddeley *et al.* , 2012 ; Nowrangi and Rosenberg, 2015 ). The source of these hippocampal–fornix–thalamic inputs is the subiculum ( Aggleton *et al.* , 1986 ), a region that shows pronounced cell loss and neurofibrillary tangle formation in Alzheimer’s disease ( Hyman *et al.* , 1990 ). The result is a disconnection of the hippocampal formation in Alzheimer’s disease, which presumably includes its projections to the anterior thalamic nuclei. Such disconnections are likely to disrupt episodic memory ( Tsivilis *et al.* , 2008 ).

This conventional description of Alzheimer’s disease still assumes that thalamic dysfunctions are a secondary response to medial temporal lobe pathologies, a chronology that may underestimate the extent to which thalamic dysfunction might initiate cognitive loss. This same standard view overlooks the potential importance of the direct projections from the limbic thalamus to the hippocampal formation ( Amaral and Cowan, 1980 ; DeVito, 1980 ), as well as the many indirect projections via the cingulate cortices ( Vann *et al.* , 2009 ). Included among these thalamic efferents to the hippocampus are those from nucleus reuniens, a nucleus of growing interest as its reciprocal hippocampal–prefrontal links appear to support learning ( Prasad and Chudasama, 2013 ; Xu and Südhof, 2013 ).

Finally, more recent post-mortem studies indicate that anterior thalamic pathology is present in other tau and TAR DNA binding protein forms of dementia ( Hornberger *et al.* , 2012 ; Tan *et al.* , 2014 ). Anterior thalamic atrophy was observed in both the semantic variant of primary progressive aphasia (semantic dementia) and the behavioural variant of frontotemporal dementia, though surprisingly, significant volume reductions were not seen in the Alzheimer’s disease cases in that study ( Hornberger *et al.* , 2012 ) compared to age-matched controls. Nevertheless, the extent of overall disruption to Papez circuit was seen as a predictor of the status of episodic memory in these dementias ( Hornberger *et al.* , 2012 ; Tan *et al.* , 2014 ), which can mimic the episodic memory deficits often seen in Alzheimer’s disease.

## Thalamic neuroimaging signatures: sporadic Alzheimer’s disease

Structural MRI provides insights into the status of the thalamus during the progression of Alzheimer’s disease. Such imaging studies are, however, problematic as the thalamus is composed of multiple nuclei, many of which cannot be safely distinguished with MRI. Should Alzheimer’s disease or its prodromal stages principally affect a small subset of thalamic nuclei, as post-mortem studies indicate, misleading null results are likely to occur. Furthermore, volume changes may best reflect late stages in the progression of neural dysfunction in Alzheimer’s disease, adding to the insensitivity of the approach. Considering these limitations, it is remarkable that thalamic changes are often described in structural MRI studies.

Given the goals of this review, priority is given to studies of MCI and its conversion to Alzheimer’s disease. Reductions of thalamic volume are typically observed in cases of amnestic MCI ( Chételat *et al.* , 2005 ; Sorg *et al.* , 2007 ; Pedro *et al.* , 2012 ; Yi *et al.* , 2015 ). A stepwise decline in thalamic status going from controls, to amnestic MCI, to Alzheimer’s disease cases was also reported in an analysis of thalamic tissue texture ( de Oliveira *et al.* , 2010 ). Likewise, MRI-based measurements show that overall thalamic volume correlates with cognitive status in MCI ( Pedro *et al.* , 2012 ; Yi *et al.* , 2015 ). Furthermore, reduced thalamic volume is found in Alzheimer’s disease cases when compared to other non-dementing individuals who reported memory lapses ( de Jong *et al.* , 2008 ), with thalamic volume again correlating with the decline in global cognitive performance. Although one study found that thalamic atrophy in MCI was not specifically associated with the risk of conversion to Alzheimer’s disease ( Yi *et al.* , 2015 ), this null result is confounded by its grouping together of diverse thalamic nuclei.

It is evident that future studies need to target specific groups or, ideally, individual thalamic nuclei. One such study looked for MRI hyperdensities in the rostral thalamus ( Swartz and Black, 2006 ), focusing on the anterior thalamic nuclei. Thalamic abnormalities occurred at raised frequencies in a variety of dementias, as well as in cases who were cognitively impaired but had no current diagnosis of dementia. Anterior medial thalamic hyperdensities were particularly associated with sudden onset or sudden decline in cognitive abilities ( Swartz and Black, 2006 ). Another analysis focussed on the structure and connectivity of ‘non-specific’ thalamic nuclei in Alzheimer’s disease ( Zarei *et al.* , 2010 ). Shape analysis revealed significant atrophy in the medial thalamus, with apparent connectivity changes in the anterodorsal thalamus as well as atrophy of the internal medullary lamina ( Zarei *et al.* , 2010 ). These changes are informative as it is within the medial thalamus that the mediodorsal nucleus, nucleus reuniens, and the anterior thalamic nuclei are found, sites that are presumed to support prefrontal cortex function via their dense reciprocal cortical connections ( Ray and Price, 1993 ; Xiao and Barbas, 2002 *a* , *b* ; Cross *et al.* , 2012 ; Prasad and Chudasama, 2013 ; Wright *et al.* , 2013 ). These pathologies within the medial thalamic region may also help to explain why some prodromal cases predominantly display deficits in executive function ( Reinvang *et al.* , 2012 ), deficits that are assumed to arise from prefrontal dysfunctions. In addition, some amnestic MCI patients show lesions in the anterior thalamic radiations associated with apathy ( Torso *et al.* , 2015 ). Such lesions would potentially disconnect the anterior thalamic nuclei, midline thalamic nuclei, and mediodorsal thalamic nucleus from the prefrontal cortex, presumably contributing to their symptoms.

A further imaging approach has used resting-state functional MRI activity to look for changes in inter-area connectivity. Such studies of MCI patients report evidence of reduced connectivity between the thalamus and multiple cortical areas ( Wang *et al.* , 2012 ; Cai *et al.* , 2015 ), including components of the ‘default mode network’ (see below). At the same time, signals of increased connectivity between the left and right thalamus have been interpreted as compensatory mechanisms ( Wang *et al.* , 2012 ; Cai *et al.* , 2015 ). Additional comparisons between early and late stage amnestic MCI patients ( Cai *et al.* , 2015 ) point to progressive, disruptive effects on various thalamic networks, including thalamo–hippocampal, thalamo–temporal, and thalamo–default mode networks ( Cai *et al.* , 2015 ).

## Thalamic neuroimaging signatures: presymptomatic at-risk Alzheimer’s disease

The realization that Alzheimer’s disease has an extended prodromal phase, possibly lasting decades ( Jack *et al.* , 2013 ), makes it desirable to examine the status of the thalamus in healthy people at heightened genetic risk of Alzheimer’s disease. At first glance, this logic might be questioned because the experiments look for thalamic abnormalities when there is no corresponding disruption of memory, as the cases are ‘presymptomatic’. In fact, the emerging view is that memory loss in these ‘presymptomatic’ stages is often obscured by ones ‘cognitive reserve’ ( Amieva *et al.* , 2014 ). Furthermore, ‘presymptomatic’ patients could have more subtle memory deficits that do not reach the threshold for a full-blown memory problem in standard neuropsychological tests. Damage to sites such as the anterior thalamus could, therefore, act as a tipping point for when symptoms of Alzheimer’s disease first appear ( Swartz and Black, 2006 ).

One valuable approach comes from the study of presenilin 1 ( *PSEN* ) mutation carriers. Studies using PET-based markers have reported increased amyloid load in the thalamus in presymptomatic presenilin 1 cases ( Knight *et al.* , 2010 ). Other imaging studies of presenilin 1 have reported that the earliest sites of amyloid deposition are the thalamus and striatum ( Ryan *et al.* , 2013 ). Reduced thalamic volume (but not hippocampal volume) was also noted in these same presymptomatic cases, along with evidence of thalamic white matter changes ( Ryan *et al.* , 2013 ). Other studies of presymptomatic carriers of familiar Alzheimer’s disease mutations have also reported atrophy in the thalamus, as well as the striatum, while temporal lobe changes appear less consistent ( Lee *et al.* , 2013 ). One potential issue is that these findings have not yet been confirmed by post-mortem examination. Another concern is that apparently excessive amyloid deposition can be observed among cognitively normal elderly subjects ( Aizenstein *et al.* , 2008 ).

A related source of evidence comes from comparing healthy carriers of the apolipoprotein ( *APOE* ) ϵ4 allele, which increases the risk of Alzheimer’s disease, with *APOE* ϵ2 allele carriers, which protect against Alzheimer’s disease ( Corder *et al.* , 1993 ). Carriers of the *APOE* ϵ4 allele show disruptions of the default mode network, which involves the posterior cingulate region ( Pihlajamäki *et al.* , 2010 ; Patel *et al.* , 2013 ). The implication is that such disruptions render people more susceptible to the advance of Alzheimer’s disease. Meanwhile, increased functional connectivity involving the thalamus has been reported in *APOE* ϵ2 carriers ( Patel *et al.* , 2013 ), which confers protection from Alzheimer’s disease. Further support for this network explanation comes from evidence that *APOE* ϵ2 carriers have increased white matter integrity in a number of sites, including the anterior thalamic radiations and the right thalamus, as well as the posterior cingulum bundle ( Chiang *et al.* , 2012 ), which innervates the posterior cingulate region and the anterior thalamic nuclei ( Mufson and Pandya, 1984 ). Meanwhile, changes in parahippocampal white matter were not found in the same study ( Chiang *et al.* , 2012 ).

Volumetric analyses of *APOE* ϵ4 carriers (increased risk of Alzheimer’s disease) have focused on the medial temporal lobes, where reduced hippocampal volumes are sometimes, but not always, found ( Adamson *et al.* , 2010 ; O’Dwyer *et al.* , 2012 ; Li *et al.* , 2014 ). While thalamic volume need not appear to alter in these at-risk cases ( O’Dwyer *et al.* , 2012 ), these null results are constrained by treating the thalamus as a single structure. Finally, the hippocampus has been used as a ‘seed’ for functional connectivity analyses of Papez circuit in *APOE* ϵ4 carriers ( Li *et al.* , 2014 ). In comparisons with non-carriers, reduced hippocampal functional connectivity was found in a number of sites, including the thalamus, while hippocampal functional connectivity correlated with episodic memory performance ( Li *et al.* , 2014 ). These findings can readily be interpreted as a disruption of Papez circuit, with consequent effects on episodic memory ( Aggleton and Brown, 1999 ).

## Anterior thalamic–retrosplenial cortex interactions

Within the context of Alzheimer’s disease symptomology, the potential importance of interactions between the anterior thalamic nuclei and retrosplenial cortex has already been stressed ( Fig. 1 ). The retrosplenial cortex (areas 29 and 30) comprises part of the posterior cingulate region, which also contains areas 23 and 31. The anterior thalamic nuclei have a particularly close affinity with the posterior cingulate region, which is most pronounced for retrosplenial cortex ( Vann *et al.* , 2009 ). In both primate and rodent brains there are dense, reciprocal connections linking retrosplenial cortex with the anterior thalamic nuclei and the laterodorsal nucleus ( Vogt *et al.* , 1987 ; Morris *et al.* , 1999 ; Aggleton *et al.* , 2014 ). Reflecting this close relationship, disconnection studies with rats show that the anterior thalamic nuclei and retrosplenial cortex function in an interdependent manner for spatial learning ( Sutherland and Hoesing, 1993 ; Dumont *et al.* , 2010 ). Although there is a need to study other forms of learning, spatial memory has often proved to be a valuable marker for hippocampal involvement in episodic-like memory in animals ( Aggleton and Pearce, 2001 ).

The strongest indication of the potential importance of retrosplenial cortex dysfunction in Alzheimer’s disease comes from PET, as well as functional MRI studies, which have repeatedly shown posterior cingulate hypoactivity in MCI and Alzheimer’s disease ( Minoshima *et al.* , 1997 ; Nestor *et al.* , 2003 *a* , *b* ; Buckner *et al.* , 2005 ; Drzezga *et al.* , 2011 ). Of particular significance was the realization that this hypoactivity, typically centred in the retrosplenial cortex, is often the first metabolic change to be detected in MCI and Alzheimer’s disease, i.e. prior to changes in the medial temporal lobe ( Minoshima *et al.* , 1997 ; Nestor *et al.* , 2003 *b* ). Intriguingly, comparisons of metabolic activity between cases with MCI and those with mild Alzheimer’s disease revealed that the latter (Alzheimer’s disease cases) displayed additional hypometabolism in the amgydala, temporoparietal and frontal association cortices. Consequently, this comparison reveals a core network of hypometabolic limbic structures (hippocampal, medial thalamus, mammillary bodies, posterior cingulate, including retrosplenial cortex) particularly associated with MCI ( Nestor *et al.* , 2003 *a* ).

Subsequent structural MRI research has revealed volume changes in the posterior cingulate region in amnestic MCI and the early stages of Alzheimer’s disease ( Scahill *et al.* , 2002 ; Chételat *et al.* , 2005 ; Frisoni *et al.* , 2009 ; Pengas *et al.* , 2010 ), though not in behavioural variant frontotemporal dementia ( Tan *et al.* , 2013 ). The descriptions of MCI cases show that posterior cingulate (including retrosplenial cortex) atrophy is present from the earliest clinical stages of Alzheimer’s disease, indicating that this region is as vulnerable as the hippocampus ( Frisoni *et al.* , 2009 ; Pengas *et al.* , 2010 ). Furthermore, the volume of the posterior cingulate region, as well as hippocampal volume, helps to predict the conversion of MCI to Alzheimer’s disease ( Chételat *et al.* , 2005 ). The implication is that pathological changes associated with retrosplenial cortex hypoactivity may occur prior to those in the medial temporal lobe, that is, in a cortical region with especial affinity for the anterior thalamic nuclei.

The posterior cingulate region, including the retrosplenial cortex, is a key component of the default mode network ( Greicius *et al.* , 2003 ; Raichle, 2015 ). This network, which shows greater activity during resting states than during many cognitive tasks, involves medial prefrontal, parietal and medial temporal lobe areas ( Raichle, 2015 ). Two thalamic sites strongly interconnected with this network are the anterior nuclei and the dorsomedial nucleus. It is, therefore, notable that a lesion in the left anterior thalamus disrupted the posterior cingulate portion of the default mode network, ipsilateral to the pathology ( Jones *et al.* , 2011 ). The relevance of this finding comes from the discovery that the default mode network is affected in amnestic MCI and Alzheimer’s disease, with changes in the network predicting conversion of MCI to Alzheimer’s disease ( Greicius *et al.* , 2003 ; Sorg *et al.* , 2007 ; Petrella *et al.* , 2011 ; Wang *et al.* , 2013 ). These default mode network changes in MCI and Alzheimer’s disease consistently involve the posterior cingulate/retrosplenial areas ( Greicius *et al.* , 2003 ; Sorg *et al.* , 2007 ) i.e. those sites most closely linked with the anterior thalamic nuclei. Indeed, studies of MCI patients reveal that the connectivity changes in this disorder involve the thalamus ( Wang *et al.* , 2013 ; Zhou *et al.* , 2013 ). Furthermore, default mode correlations with memory performance in MCI have been found for the thalamus, as well as the hippocampus ( Wang *et al.* , 2013 ). Thus, although research on the default mode network and its disruption in Alzheimer’s disease has concentrated on cortical mechanisms, there are good grounds to suppose that thalamic dysfunctions contribute to these changes.

Studies with animals have more precisely examined anterior thalamic–retrosplenial cortex relationships, i.e. targeted this link within Papez circuit. In a series of rodent studies, anterior thalamic lesions consistently produced chronic dysfunctions in retrosplenial cortex ( Aggleton and Nelson, 2015 ). These dysfunctions included disrupted gene transcription, e.g. a loss of immediate-early gene expression, as well as reduced metabolic activity and a loss of some forms of neuronal plasticity ( Van Groen *et al.* , 1993 ; Jenkins *et al.* , 2004 ; Poirier *et al.* , 2008 ; Garden *et al.* , 2009 ; Dumont *et al.* , 2012 ; Mendez-Lopez *et al.* , 2013 ). Consequently, there are good grounds to believe that the integrity of the anterior thalamic nuclei is vital for retrosplenial cortex activity and function. The extent of this structural interdependence can be widened further as anterior thalamic lesions also disrupt markers of neuronal plasticity in the hippocampus ( Jenkins *et al.* , 2002 ; Dumont *et al.* , 2012 ). The implication is that these limbic structures function in a network that depends on the integrity of all components.

Clinical studies of anterograde amnesia reveal that both the retrosplenial cortex and anterior thalamic nuclei are vital for episodic memory ( Aggleton and Brown, 1999 ; Harding *et al.* , 2000 ; Maguire, 2001 ; Vann *et al.* , 2009 ; Carlesimo *et al.* , 2011 ). Such findings underline the potential impact of disturbances to anterior thalamic–posterior cingulate interactions during the progression of Alzheimer’s disease. However, very few studies have explored how retrosplenial-thalamic changes might affect clinical presentation. In a recent exception, patients with Alzheimer’s disease were shown to be particularly impaired when asked to change from allocentric to egocentric spatial orientations ( Tu *et al.* , 2015 ), a difficulty that correlated strongly with retrosplenial/posterior cingulate atrophy in the same patients. This result closely matches animal findings, which indicate that spatial orientation changes require the concerted actions of the hippocampus, posterior cingulate cortex, and anterior thalamus, with the retrosplenial cortex being the hub for these interactions ( Byrne *et al.* , 2007 ; Vann *et al.* , 2009 ; Nelson *et al.* , 2015 ). This comparative approach provides a promising avenue for future research on thalamic-retrosplenial interactions.

Unfortunately, one class of evidence largely lacking at present concerns the impact of retrosplenial cortex dysfunction upon anterior thalamic activity, i.e. whether cortical pathologies automatically lead to thalamic abnormalities. It is, however, known that retrosplenial lesions in rats result in both gliosis and cell loss in the anterior thalamic nuclei, neuropathologies that are most evident in the medial part of the anteroventral thalamic nucleus ( Neave *et al.* , 1994 ). Likewise, hippocampal lesions in rats reduce immediate-early gene activity in the anterior thalamic nuclei and retrosplenial cortices ( Jenkins *et al.* , 2006 ; Albasser *et al.* , 2007 ). Another intriguing piece of evidence for the interdependency of these sites comes from a patient with retrosplenial damage associated with anterograde amnesia ( Heilman *et al.* , 1990 ). Measurements of metabolic activity (PET) revealed thalamic hypoactivity, but no apparent medial temporal lobe changes ( Heilman *et al.* , 1990 ).

From a neurodegenerative perspective, patients with *C9orf72* mutations might be of particular relevance when investigating structural relationships in Alzheimer’s disease. Such *C9orf72* patients belong to the frontotemporal dementia–amyotrophic lateral sclerosis spectrum, which usually affects more prefrontal, motor and temporal cortices. However, *C9orf72* patients present with significant parietal (including retrosplenial cortex) and thalamic atrophy ( Mahoney *et al.* , 2012 ; Whitwell *et al.* , 2012 ; Irish *et al.* , 2013 ). Interestingly, these changes have been associated with more significant episodic memory problems, despite the patients having relatively intact medial temporal lobes ( Irish *et al.* , 2013 ). Thus, future contrasts of *C9orf72* and Alzheimer’s disease might be of particular interest in delineating the functions of the thalamic-retrosplenial axis and their potential role in Alzheimer’s disease symptomology. These contrasts should be corroborated by more formal experiments to detail anterior thalamic–retrosplenial interactions in animal models of Alzheimer’s disease.

## Animal models

Genetically modified animals offer an alternative means to study the progression of particular pathological features in Alzheimer’s disease. These models often involve the abnormal production of amyloid plaques, tau neurofibrils, or both. One of the most important reasons for using animal models is to provide the anatomical specificity that would otherwise be unobtainable in humans. Unfortunately, this opportunity has often been wasted when it comes to the thalamus. Instead, there has been considerable focus on the status of the hippocampal formation in these models. Consequently, current findings concerning the thalamus remain sketchy, despite the large number of animal studies that have adopted this general approach.

One of the first mouse models to be examined was the Tg2576 APP (swe) strain, which shows excessive deposition of amyloid-β ( Hsiao *et al.* , 1996 ). Studies of this mouse have repeatedly focused on the hippocampal formation, but one set of analyses principally examined retrosplenial cortex ( Poirier *et al.* , 2011 ). That study reported clear changes in markers for neuronal activity and metabolism in the retrosplenial cortex long before evidence of plaque formation in the brain ( Poirier *et al.* , 2011 ). Related analyses showed that subsequent amyloid-β deposition in the anterior thalamic nuclei was closely associated with amyloid-β deposition in the retrosplenial cortex ( Poirier *et al.* , 2011 ). Other evidence comes from a double mutant strain with excessive amyloid-β deposition (APPswe/PS1dE9), in which seizures become apparent at the time of plaque formation ( Gurevicius *et al.* , 2013 ). Changes in both thalamic and cortical excitability (local field potentials and EEG) were present ( Gurevicius *et al.* , 2013 ).

In a third mouse line (TgaPParc), diffuse amyloid-β deposition first appears in the subiculum, with some animals also showing early changes in retrosplenial cortex ( Rönnbäck *et al.* , 2012 ). Amyloid changes then occur in the mammillary bodies and thalamus, followed later by the dentate gyrus and hippocampal CA fields. These results were interpreted as a pattern of spread along Papez circuit, starting in the subiculum ( Rönnbäck *et al.* , 2012 ). Unfortunately, the precise thalamic nuclei with excessive amyloid deposition were not specified ( Rönnbäck *et al.* , 2012 ). In a follow-up study of this same genetic model, subiculum lesions reduced amyloid levels in CA1 and the retrosplenial cortex ( George *et al.* , 2014 ). While these results are consistent with a pattern of spread emanating from the subiculum, that study ( George *et al.* , 2014 ) did not include data for the anterior thalamus.

In a mouse model that combines both amyloid-β and tau pathology, significant neuronal loss occurs in the subiculum and hippocampus ( Wilcock *et al.* , 2008 ), so mimicking additional features of human Alzheimer’s disease. Immunohistochemical analyses revealed particularly dense amyloid-β staining in the subiculum and thalamus in this same mouse model. In addition, hyperphosphorylation of tau occurred in the same subiculum and thalamic locations ( Wilcock *et al.* , 2008 ). Unfortunately, the report did not specify the precise thalamic location of these pathological changes ( Wilcock *et al.* , 2008 ).

It is evident that future studies of such models should take full advantage of the anatomical precision made possible by animal studies. Advances include the visualization of thalamic amyloid-β plaques using high-field scanning methods ( Faber *et al.* , 2007 ). A key goal will be to provide longitudinal analyses of individual thalamic nuclei, relating any cognitive changes to those seen in temporal, parietal, and frontal lobe areas. While it is inevitable that the focus will be on behavioural measures of memory loss, research using broader test batteries have revealed additional deficits in attention and response control in transgenic models of Alzheimer’s disease ( Romberg *et al.* , 2013 ).

## Clinical and therapeutic implications

The present review has amassed evidence that the limbic thalamus forms a critical hub for episodic memory and spatial orientation and, therefore, could impact on the symptomology in Alzheimer’s disease and its prodromal phases. As outlined above, the consequences of thalamic dysfunction in individual thalamic nuclei are barely recognized in accounts of Alzheimer’s disease, a situation that needs correcting. More specifically, clinicians should not only take account of hippocampal atrophy on clinical scans but also consider concurrent anterior thalamic and retrosplenial changes. Previous evidence (e.g. Hornberger *et al.* , 2012 ) has shown that thalamic changes can have an additional impact on episodic memory, even when the hippocampi are grossly affected by neurodegenerative pathology. One priority would be to develop specific cognitive measures that tap into anterior thalamic dysfunction. Such tests might then be used as outcome measures in therapeutic disease intervention trials.

Some guidance for these goals comes from clinical and behavioural studies that have provided new insights into how anterior thalamic activity may contribute to memory. Recent *in vivo* intra-thalamic recordings in volunteer patients suffering from epilepsy ( Sweeney-Reed *et al.* , 2014 , 2015 ) have revealed correlates between anterior thalamic activity and learning. In particular, neocortical–anterior thalamic nuclei synchrony (theta-gamma cross-frequency coupling) predicted subsequent memory performance ( Sweeney-Reed *et al.* , 2014 ). Successful memory encoding was also related to theta phase alignment in the anterior thalamic nuclei ( Sweeney-Reed *et al.* , 2015 ). Meanwhile, recording studies in rats have uncovered neurons in the anterior thalamic nuclei that show spatial firing reflecting different aspects of location and navigation, indicative of high-resolution information processing ( Jankowski *et al.* , 2015 ).

At the same time, both recent clinical and behavioural studies are starting to reveal that the anterior thalamic nuclei have additional roles, e.g. in regulating attention. It is presumed that these roles partly reflect their dense, reciprocal connections with prefrontal and parietal cortices. An analysis of stroke patients found that pathologies involving the anterior and ventrolateral thalamus disrupt the interplay between working memory and attention ( de Bourbon-Teles *et al.* , 2014 ). A complementary behavioural study highlighted a role for the rat anterior thalamic nuclei in guiding attention to task-relevant stimuli ( Wright *et al.* , 2015 ). For these reasons, it would be informative to look for changes in both episodic memory and executive function, including attention based on learnt contingencies, when using cognitive assays of Alzheimer’s disease and its prodromal conditions.

Such analyses build on considerable evidence that both Alzheimer’s disease and MCI are associated with losses in some forms of attention ( Perry and Hodges, 1999 , 2003 ; Belleville *et al.* , 2007 ). Tests of divided attention and set-shifting are disrupted in early Alzheimer’s disease ( Perry and Hodges, 1999 ; Perry *et al.* , 2000 ), with related studies indicating that MCI impairs top-down attentional control, while sparing attentional dwell time ( Perry and Hodges, 2003 ; Belleville *et al.* , 2007 ). At the same time, functional MRI evidence from attentional tasks points to changes in networks linked with prefrontal cortex in cases of MCI and Alzheimer’s disease ( Dannhauser *et al.* , 2005 ; Hao *et al.* , 2005 ). Tests of set-formation and set-shifting are of particular interest as they are disrupted in early Alzheimer’s disease ( Perry *et al.* , 2000 ) and have recently been linked to anterior thalamic function ( de Bourbon-Teles *et al.* , 2014 ; Wright *et al.* , 2015 ). While set-shifting is closely tied to prefrontal and parietal functions ( Dias *et al.* , 1996 ; Rushworth *et al.* , 2001 ), the hippocampus appears far less critical for this function ( Milner *et al.* , 1968 ; Owen *et al.* , 1991 ). This pattern of results points to the value of examining whether thalamic neuropathology is related to the attentional changes seen in MCI and Alzheimer’s disease, with the appreciation that there are different subtypes of each disorder, reflecting different patterns of memory loss ( Petersen, 2004 ). A further consideration is the way in which attentional deficits might impact on memory.

It is important to stress that the significance of the anterior thalamic nuclei for cognition arises from their being both upstream and downstream of the hippocampus ( Aggleton and Brown, 1999 ; Vann, 2013 ; Fig. 1 ). One potential application, which reflects their upstream status, arises from the way that gradual neurodegenerative processes may leave viable, residual medial temporal lobe tissue for a long proportion of time during the disease process. This situation creates the possibility that thalamic stimulation might maintain or even boost memory and attention. Indeed, it is known that in both rats and mice, high frequency electrical stimulation of the anterior thalamic nuclei can increase hippocampal neurogenesis ( Toda *et al.* , 2008 ; Encinas *et al.* , 2011 ; Hamani *et al.* , 2011 ; Zhang *et al.* , 2015 ). This same treatment can help to improve spatial learning in rats ( Encinas *et al.* , 2011 ; Zhang *et al.* , 2015 ). In a further study, amyloid-β _1-40_ was first injected into the hippocampus of rats, followed by electrical stimulation of the anterior thalamic nuclei ( Chen *et al.* , 2014 ). This treatment improved spatial learning in these compromised rats. One potential limitation with these rodent stimulation studies is that the anterior thalamic nuclei remain intact, while in cases of MCI or Alzheimer’s disease the staging of the neuropathological changes is such that thalamic dysfunction could compromise the efficacy of such an approach.

Even so, a related procedure has been attempted in human volunteers. This approach involved chronic deep brain stimulation in the region of the fornix in patients with mild, probable Alzheimer’s disease ( Laxton *et al.* , 2010 ; Smith *et al.* , 2012 ). Patients received continuous stimulation for 12 months. Imaging studies (functional MRI and PET) showed that the stimulation activated the default mode network and drove neural activity in the hippocampus ( Laxton *et al.* , 2010 ). Increases in glucose utilization were found in frontal, temporal, and parietal networks that involved the thalamus ( Smith *et al.* , 2012 ). The same procedure was associated with improved outcomes for global cognition, memory, and quality of life ( Smith *et al.* , 2012 ). The location of the fornix stimulating electrodes is of particular relevance as they were placed in the hypothalamus, close to the posterior commissural division of the fornix. This component of the fornix principally innervates the mammillary bodies ( Poletti and Creswell, 1977 ). If the deep brain stimulation activated the fornix, as assumed ( Laxton *et al.* , 2010 ), it would not only directly activate the mammillary bodies but would also indirectly activate the anterior thalamic nuclei, via the mammillothalamic tract ( Vann and Aggleton, 2003 ; Vann *et al.* , 2007 ; Dillingham *et al.* , 2015 ). The status of the mammillothalamic tract has been strongly associated with episodic memory function ( Van der Werf *et al.* , 2003 ; Carlesimo *et al.* , 2011 ; Danet *et al.* , 2015 ).

Future investigations should increasingly consider the interactions of tau and amyloid pathology across Papez network. The rationale for such an approach is supported by a recent study ( Khan *et al.* , 2014 ) showing that entorhinal dysfunction is better explained by the interaction of tau and amyloid pathology than either separately. The advent of tau-ligand PET imaging has further corroborated this picture, showing that pathological interactions of tau and amyloid can better explain disease progression and symptomology in Alzheimer’s disease than either individual pathology ( Khan *et al.* , 2014 ). Khan and colleagues (2014 ) also showed that tau-amyloid interaction not only affected the entorhinal cortex but also influenced its connectivity to the parietal cortex and, thus potentially, posterior cingulate and retrosplenial areas. A similar scenario can be imagined for the thalamus, in which combined tau–amyloid interactions explain the emergence of dysfunctions in this region better than simple correlations with amyloid or tau *per se* .

It remains the case, however, that current analyses typically take a hippocampal-centred view, with medial temporal lobe changes seen to instigate mnemonic dysfunctions. While this view might prove to be correct, it is surely necessary to test alternative models of the neuropathological progression in Alzheimer’s disease. In fact, the breadth and depth of evidence supports a network model ( Acosta-Cabronero and Nestor, 2014 ), which also more accurately reflects what we now know about the normal anatomical substrates for episodic memory ( Aggleton and Brown, 1999 ). Such distributed models also highlight the importance of determining white matter microstructure in those tracts linking key sites within the network, namely the fornix and cingulum bundle ( Fig. 1 ) ( Acosta-Cabronero and Nestor, 2014 ). This broader orientation also provides a basis from which to compare those dementias with different pathologies, yet are able to afflict memory in apparently similar ways ( Frisch *et al.* , 2013 ). The same body of evidence points to the real possibility that a break down in anterior thalamic– retrosplenial interactions might be the starting point for the characteristic, early loss of episodic memory in prodromal Alzheimer’s disease, with thalamic dysfunctions occurring at the same time, or even before, those in the hippocampus. A related view is that thalamic pathology may provide a tipping point for when medial temporal lobe dysfunctions become symptomatic.

This review challenges prevalent views about the genesis of Alzheimer’s disease. Two principal issues are: (i) whether hippocampal pathology is sufficient to explain the loss of episodic memory in MCI and Alzheimer’s disease; and (ii) whether thalamic pathology is necessary for these cognitive changes. While it is not yet possible to be conclusive on either issue, there is considerable evidence that thalamic dysfunctions in sites critical for episodic memory are an early (presymptomatic) feature of the disorder. It is also apparent that there are subtypes of Alzheimer’s disease that differ in their relative extent of hippocampal pathology ( Whitwell, *et al.* , 2012 ). Furthermore, it is striking that cases of semantic dementia and Alzheimer’s disease can appear to have comparable levels of medial temporal hypoactivity, yet differ markedly in their episodic memory status ( Nestor *et al.* , 2006 ). While the additional semantic memory loss in semantic dementia was associated with anterior temporal cortex hypometabolism, the additional episodic memory loss in Alzheimer’s disease was associated with dysfunctions in the diencephalic (mammillary bodies, medial thalamus) and posterior cingulate (including retrosplenial) network ( Nestor *et al.* , 2006 ). Meanwhile, studies of diencephalic amnesia have repeatedly shown that overt hippocampal pathology is not a necessary condition for severe episodic memory loss. Taken together, there is a strong case for a detailed, systematic approach to the contributions of the limbic thalamus to the progression of memory loss in MCI and Alzheimer’s disease, in which an open mind is kept concerning the primacy of the hippocampus.

## Funding

The authors wish to thank the Wellcome Trust (grant #103722/Z14/Z) for supporting research that promoted this review.

## Abbreviation

MCI =: mild cognitive impairment
